# Effects of caffeine intake on muscle strength and power: a systematic review and meta-analysis

**DOI:** 10.1186/s12970-018-0216-0

**Published:** 2018-03-05

**Authors:** Jozo Grgic, Eric T. Trexler, Bruno Lazinica, Zeljko Pedisic

**Affiliations:** 1 Institute of Sport, Exercise and Active Living (ISEAL), Victoria University, Melbourne, Australia; 2 Applied Physiology Laboratory, Department of Exercise and Sport Science, University of North Carolina, Chapel Hill, NC USA; 3 Human Movement Science Curriculum, Department of Allied Health Sciences, University of North Carolina, Chapel Hill, NC USA; 4 Faculty of Education, Department of Kinesiology, J.J. Strossmayer University, Osijek, Croatia

**Keywords:** Ergogenic aid, Performance, Power, Data synthesis

## Abstract

**Background:**

Caffeine is commonly used as an ergogenic aid. Literature about the effects of caffeine ingestion on muscle strength and power is equivocal. The aim of this systematic review and meta-analysis was to summarize results from individual studies on the effects of caffeine intake on muscle strength and power.

**Methods:**

A search through eight databases was performed to find studies on the effects of caffeine on: (i) maximal muscle strength measured using 1 repetition maximum tests; and (ii) muscle power assessed by tests of vertical jump. Meta-analyses of standardized mean differences (SMD) between placebo and caffeine trials from individual studies were conducted using the random effects model.

**Results:**

Ten studies on the strength outcome and ten studies on the power outcome met the inclusion criteria for the meta-analyses. Caffeine ingestion improved both strength (SMD = 0.20; 95% confidence interval [CI]: 0.03, 0.36; *p* = 0.023) and power (SMD = 0.17; 95% CI: 0.00, 0.34; *p* = 0.047). A subgroup analysis indicated that caffeine significantly improves upper (SMD = 0.21; 95% CI: 0.02, 0.39; *p* = 0.026) but not lower body strength (SMD = 0.15; 95% CI: -0.05, 0.34; *p* = 0.147).

**Conclusion:**

The meta-analyses showed significant ergogenic effects of caffeine ingestion on maximal muscle strength of upper body and muscle power. Future studies should more rigorously control the effectiveness of blinding. Due to the paucity of evidence, additional findings are needed in the female population and using different forms of caffeine, such as gum and gel.

## Background

Caffeine’s ergogenic potential has been extensively studied in the sports science literature, with research dating back to 1907 [1]. From investigating caffeine’s effects on aerobic exercise, in recent years the research focus has shifted to anaerobic exercise performance outcomes, such as muscular endurance, muscle strength, and jumping tasks that require muscle power. While caffeine has been found to significantly enhance muscular endurance [2], the effects of caffeine ingestion on maximal muscle strength (commonly operationalized as one repetition maximum [1RM]) and muscle power (commonly operationalized as vertical jump) remain unclear, and the practical utility of caffeine ingestion for enhancing performance in such physical tasks has not been fully elucidated.

The pioneering work on caffeine’s effects on strength by Astorino et al. [3] reported no significant strength-enhancing effects with caffeine ingestion in a group of resistance trained men. Recent work by Grgic and Mikulic [4], however, found a significant 3% increase in lower body strength with caffeine ingestion using the barbell back squat 1RM as a measure of maximal strength. Goldstein et al. [5] reported a significant increase in upper body strength with caffeine ingestion, while Williams et al. [6] reported no ergogenic effect. The inconsistent results of individual studies prevent drawing sound conclusions regarding the ergogenic potential of caffeine for maximal strength outcomes.

Equivocal findings have also been presented for the effects of caffeine intake on muscle power. A recent study by Ali et al. [7] reported no effect on countermovement jump height with caffeine ingestion. However, the findings of Bloms et al. [8] support conclusions about caffeine as an effective ergogenic aid for achieving acute improvements in countermovement jump height and peak force. Given the importance of jumping abilities for many common sports, it would be of both scientific and practical significance to determine a reasonably precise estimate regarding the potential performance-enhancing impact of caffeine ingestion on muscle power.

Several aspects that vary between studies, including the exercise used, participants’ characteristics (e.g., age, sex, and training experience), and caffeine form, might be responsible for the inconsistency of findings. Most importantly, small sample sizes often limited the statistical power to detect significant effects [9]. A meta-analysis of individual studies is needed to circumvent these issues and provide in-depth, evidence-based scrutiny of the current body of evidence. The first meta-analytic investigation on the topic of caffeine and strength was performed by Warren et al. [10], who found a mean increase of approximately 7% in lower body maximal voluntary contraction with caffeine ingestion. A limitation of the meta-analysis is that only two of the included studies tested the effects of caffeine ingestion on 1RM, which significantly restricted the findings to isometric and isokinetic strength outcomes.

The latest meta-analysis on the topic, done by Polito et al. [2], found no significant effect of caffeine intake on performance in 1RM strength tests. However, only three studies met the inclusion criteria for the meta-analysis. The total number of pooled participants was relatively low (*n* = 46), potentially indicating issues with the statistical power of the analysis. Furthermore, the small number of included studies prevented subgroup analyses for possible moderators that may potentially impact the ergogenic potential of caffeine. Since the review by Polito et al. [2], a number of experimental trials have been published [4, 11–16], presenting novel findings for females [14], trained [4, 16] and untrained men [11, 13], athletes [15], and adolescents [12]; as such, an updated review appears to be warranted.

No previous meta-analyses have pooled the results of individual studies on the effects of caffeine on muscle power. The aim of this systematic review was, therefore, twofold: (a) to perform an updated meta-analysis of the acute effects of caffeine ingestion on maximal muscle strength; and (b) to conduct the first meta-analysis of acute effects of caffeine ingestion on muscle power assessed by vertical jump tests. The results may benefit athletes and practitioners in a variety of sports in which muscle strength and/or power are important determinants of performance.

## Methods

### Search strategy

The systematic literature search was performed following the PRISMA guidelines [17]. A search of the following databases was performed: PubMed/MEDLINE, Scopus, Cochrane Library, Web of Science (including Science Citation Index Expanded, Social Sciences Citation Index, and Arts & Humanities Citation Index), Google Scholar, Networked Digital Library of Theses and Dissertations, ProQuest Dissertation & Theses and Open Access Theses and Dissertations. The search for the studies on the effects of caffeine on strength was restricted to the documents published from 2015 onwards as the review by Polito et al. [2], with a search performed in March 2015 was used as a reference point. The review by Polito and colleagues [2] was assessed for rigor and deemed as of high-quality. Thus, the studies [3, 5, 6] included in the work by Polito et al. [2] were also included in the present review. The following syntax was used for the primary search: caffeine AND (“muscle strength” OR “ergogenic aid” OR performance OR “resistance exercise” OR “resistance training” OR recovery OR “strength training”).

A separate search was done for the studies on the effects of caffeine on power outcomes. The following syntax with no time restriction was used: caffeine AND (“vertical jump” OR “countermovement jump” OR “squat jump” OR plyometrics OR height OR “drop jump” OR “depth jump” OR “jump training”).

The search results were downloaded and filtered in EndNote software (X8; Clarivate Analytics, New York, USA). A secondary search was performed by screening the reference lists of all selected studies, and by conducting forward citation tracking (using Google Scholar and Scopus) of studies found meeting the inclusion criteria. The search concluded on April 19th, 2017.

### Inclusion criteria

To warrant inclusion in the current analysis potential studies were required to meet the following criteria:

- an experimental trial published in English in a peer-reviewed journal, or a doctoral or a master’s thesis;
- assessed the effects of caffeine ingestion in the form of capsule, liquid, gum or gel on dynamic maximal muscle strength (i.e. the greatest amount of weight lifted in a single repetition – 1RM) using constant external resistance, and/or on muscle power assessed using a vertical jump test (both peak force and vertical jump height were considered);
- caffeine was not co-ingested with other drugs/substances or potentially ergogenic compounds;
- employed a single or double-blind, randomized crossover design;
- used human participants without known chronic disease or injury.

Studies were excluded from the analysis if any of the above criteria were violated. Caffeine ingestion via coffee was not considered as coffee has several other biologically active compounds that might moderate the impact of caffeine.

### Study coding and data extraction

For all studies meeting the inclusion criteria, the following information was tabulated on a predefined coding sheet using Microsoft Excel software (Microsoft Corporation, WA, USA):

- author(s), title and year of publication;
- sample size, participants’ sex, participants’ age (categorized as: adolescents [10–18 years]; young adults [18–39 years]; middle-aged adults [40–64 years];and seniors [≥65 years], and participants’ experience in resistance training (categorized as: untrained [less than 1 year of experience]; and trained [more than 1 year of experience]) for studies assessing strength outcomes, and experience in sport training using the same categories as above for studies assessing muscle power.
- caffeine form, dosage, and time of ingestion before the experimental session(s);
- the exercises used for assessing muscle strength and power with the accompanying mean ± standard deviation (SD) data for the placebo and caffeine trials;
- habitual caffeine intake by the participants;
- the number of participants indicating which trial they perceived to be the caffeine trial;
- reported side effects;
- reported funding for conducting the studies.

### Methodological quality

The 11-point PEDro scale was used for the assessment of the methodological quality of studies [18]. The first item concerns external validity and is not included in the total score; hence, the maximal score on the scale is 10. Studies were classified as in McCrary et al. [19]. Two authors of the article (JG and BL) performed the search, coding, and appraisal of methodological quality independently, with discussion and consensus over any observed differences. Before correcting for observed differences, the overall agreement between the two independent data extractions was very high (Cohen’s kappa = 0.94).

### Statistical analysis

The meta-analysis was performed using the Comprehensive Meta-analysis software, version 2 (Biostat Inc., Englewood, NJ, USA). Standardized mean differences (Hedge’s g [SMD]) and 95% confidence intervals (CI) were calculated between the placebo and caffeine trials based on their means and standard deviations in 1RM (kg) and vertical jump (cm) tests, the correlations between the trials, and the number of participants. An analysis of peak force in the vertical jump test was not performed as only two studies reported such outcomes [8, 16]. Since none of the studies reported correlation, a 0.5 correlation was assumed for all trials, as recommended by Follmann et al. [20]. When a study measured muscle strength and/or power under multiple conditions (e.g. used more than one caffeine dose, tested more than one muscle group), SMDs and variances were averaged across the different conditions. SMDs of ≤0.2, 0.2–0.5, 0.5–0.8, and > 0.8 were considered to represent small, medium, large and very large effects, respectively [9]. The random effects model was used for analysis of both muscle strength and muscle power outcomes. The statistical significance threshold was set a priori at *p* < 0.05.

Subgroup analyses for the effects of caffeine on muscle strength were performed for the following study characteristics: (a) upper body strength; (b) lower body strength; (c) the capsule form of caffeine; (d) the liquid form of caffeine; (e) females; (f) males; (g) untrained; and (h) trained. Subgroup analyses for the effects of caffeine on muscle power were performed for the following characteristics: (a) the capsule form of caffeine; (b) the liquid form of caffeine; (c) females; (d) males; (e) athletes; (h) non- athletes; (f) countermovement and squat jump tests; and (g) Sargent jump tests.

The *I*^2^ statistic was used to assess the degree of heterogeneity, with values from ≤50% indicating low heterogeneity, 50–75% moderate heterogeneity and > 75% high level of heterogeneity. Funnel plots were constructed for both muscle strength and muscle power outcomes, plotting standard error against Hedge’s g. Funnel plot asymmetry arising from potential publication bias was assessed using the Trim-and-Fill method [21].

## Results

**Fig. 1:**
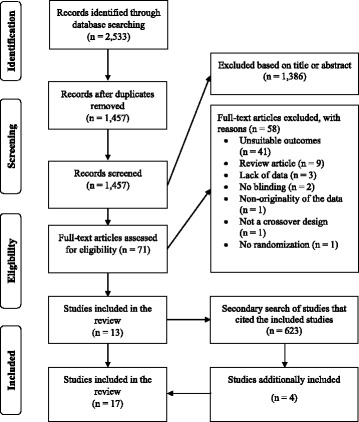
Flow diagram of the search and study selection process

The literature search yielded a total of 2533 documents. After a preliminary screening of titles and abstracts, 71 full-text studies were scrutinized. In total, ten studies were found meeting the inclusion criteria for strength outcomes [3–6, 11–16] (Table 1) with a total of 149 participants (males *n* = 116, females *n* = 33). Ten studies were found assessing muscle power outcomes [4, 7, 8, 15, 22–26] with a total of 145 participants (males *n* = 116, females *n* = 29). According to their age, all participants were classified as adolescents or young adults. Three studies [4, 12, 15] assessed both muscle strength and muscle power. The results of the search and study selection process are depicted in Fig. 1.

**Table 1:** Studies included in the analysis: summary of study designs

Study	Study design	Participants age (years)	Sample size and sex	Resistance/sport training experience	Habitual caffeine intake (mg.d^−1^)^a^	Caffeine form	Caffeine dosage (mg.kg^−1^)	Timing of caffeine ingestion before the experimental session(s) [minutes])	Exercise(s) used for the muscle strength/power assessment	PEDro score
Ali et al. [7] 2016	RDB	24 ± 4	10 females	Athletes	0–300	Capsule	6	60	CMJ	10
Andrade-Souza et al. [22] 2014	RDB	25 ± 3	11 males	Athletes	N/A	Capsule	6	60	CMJ	8
Arazi et al. [12] 2016*a*	RDB	17 ± 1	10 females	Untrained/ Athletes	< 60	Capsule	2 and 5	60	LP and ST	10
Arazi et al. [11] 2016*b*	RDB	21 ± 4	15 males	Untrained	N/A	Capsule	6	60	BP and LP	10
Astorino et al. [3] 2008	RDB	23 ± 4	22 males	Trained	110 ± 152	Capsule	6	60	BP and LP	10
Bloms et al. [8] 2016	RSB	20 ± 1	9 females	Athletes	N/A	Capsule	5	60	CMJ and SJ	8
		21 ± 2	16 males							
Brooks et al. [13] 2015	RDB	21 ± 3	14 males	Untrained	N/A	Capsule	5	60	MBS	10
Clarke et al. [23] 2016	RDB	21 ± 2	8 males	Athletes	N/A	Capsule	3	60 and during the testing sessions	CMJ	10
Diaz-Lara et al. [15] 2016	RDB	29 ± 3	14 males	Trained/ Athletes	< 60	Capsule	3	60	BP and CMJ	10
Foskett et al. [24] 2009	RDB	24 ± 5	12 males	Athletes	0–350	Liquid	6	60	CMJ	10
Gant et al. [25] 2010	RDB	21 ± 3	15 males	Athletes	N/A	Liquid	260 (fixed) 3.7 on average	60 and during the testing sessions	CMJ	10
Gauvin [26] 2016	RDB	22 ± 2	23 males	Untrained/ Non-athletes	< 200 per week	Capsule	7	60	CMJ	9
Goldstein et al. [5] 2010	RDB	25 ± 7	15 females	Trained	< 250 (*n* = 8) > 250 (*n* = 7)	Liquid	6	60	BP	10
Grgic et al. [4] 2017	RDB	26 ± 6	17 males	Trained/ Non-athletes	58 ± 92	Liquid	6	60	BP, BBS and ST	9
Martin [16] 2015	RDB	20 ± 1	12 males	Trained	N/A	Gel	75 (fixed) - 0.9 on average	60	BP and BBS	10
Sabblah et al. [14] 2015	RSB	24 ± 3	7 females	Trained	N/A	Liquid	5	60	BP and MBS	8
		28 ± 6	10 males							
Williams et al. [6] 2008	RDB	26 ± 4	9 males	Trained	‘Low’ (no exact values)	Capsule	300 (fixed) - 3.6 on average	45	BP and LPD	10

^a^intake per day unless stated otherwise; *RDB* randomized double-blind study, *RSB* randomized single-blind study, *CMJ* countermovement jump, *SJ* squat jump, *LP* leg press, *ST* Sargent test, *BP* bench press, *MBS* machine-based squat, *LPD* lat pulldown, *BBS* barbell back squat

Fifteen studies were published in peer-reviewed journals, while two studies were master’s theses [14, 26]. The median number of participants per study was 14. Most of the studies used a double-blind design (i.e., 15 studies), with two studies [8, 14] using a single-blind design. Caffeine dosage varied from 0.9 mg.kg^− 1^ to 7 mg.kg^− 1^. Only one study administered caffeine in the form of gel [16], while the rest used capsule or liquid forms. Only nine studies reported habitual caffeine intake, with Astorino et al. [3] and Goldstein et al. [5] reporting a large range of habitual caffeine intakes among the participants (0–600 mg.kg^− 1^ per day). Only three studies [3, 22, 24] reported assessing the effectiveness of the blinding, with 60%, 50% and 33% of the participants correctly differentiating between the placebo and the caffeine trials, respectively. Individual characteristics of the included studies are reported in Table 1.

**Fig. 2:**
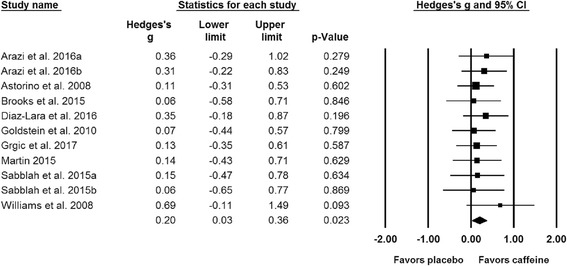
Forest plot showing differences between the effects of placebo and caffeine trials on measures of maximal muscular strength. The size of the plotted squares reflects the relative statistical weight of each study. The numbers on the *x*-axis denote the standardized mean differences expressed as Hedge’s g. The horizontal lines denote the respective 95% confidence intervals (CI)

**Fig. 3:**
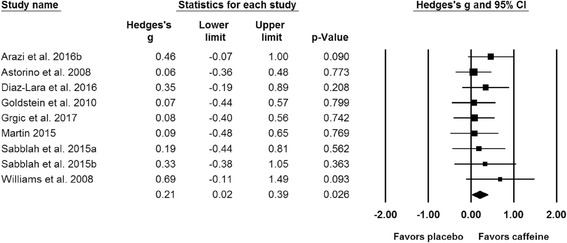
Forest plot showing differences between the effects of placebo and caffeine trials on measures of upper-body maximal muscle strength. The size of the plotted squares reflects the relative statistical weight of each study. The numbers on the *x*-axis denote the standardized mean differences expressed as Hedge’s g. The horizontal lines denote the respective 95% confidence intervals (CI)

**Fig. 4:**
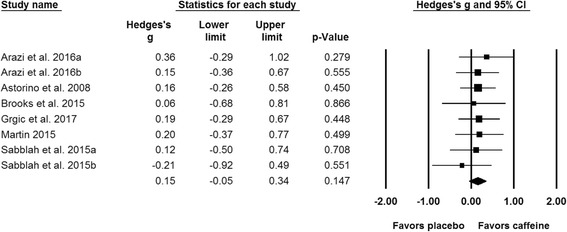
Forest plot showing differences between the effects of placebo and caffeine trials on measures of lower-body maximal muscle strength. The size of the plotted squares reflects the relative statistical weight of each study. The numbers on the *x*-axis denote the standardized mean differences expressed as Hedge’s g. The horizontal lines denote the respective 95% confidence intervals (CI)

Results of the meta-analysis indicated a significant difference (*p* = 0.023) between the placebo and caffeine trials on measures of maximal strength (Fig. 2). The pooled SMD for the effects of caffeine ingestion on muscle strength was 0.20 (95% CI: 0.03, 0.36). A subgroup analysis indicated that caffeine significantly improves upper (SMD = 0.21; 95% CI: 0.02, 0.39; *p* = 0.026; Fig. 3) but not lower body strength (SMD = 0.15; 95% CI: -0.05, 0.34; *p* = 0.147; Fig. 4). Results from all of the remaining subgroup analysis may be found in Table 2.

**Table 2:** Results from the subgroup meta-analyses

Subgroup analysis	SMD [95% CI]	*p*-value	Mean caffeine dose (mg.kg^−1^[range])
Strength outcomes			
Upper body strength	0.21 [0.02, 0.39]	0.026	4.7 [0.9–6]
Lower body strength	0.15 [−0.05, 0.34]	0.147	4.8 [0.9–6]
Capsule form of caffeine	0.27 [0.04, 0.50]	0.023	4.7 [2–6]
Liquid form of caffeine	0.11 [−0.17, 0.39]	0.462	6 [6]
Males	0.21 [0.02, 0.41]	0.034	4.7 [0.9–6]
Females	0.15 [−0.13, 0.43]	0.294	5 [2–6]
Trained participants	0.18 [−0.02, 0.37]	0.076	4.8 [0.9–6]
Untrained participants	0.27 [−0.09, 0.63]	0.144	4.8 [2–5]
Power outcomes			
Capsule form of caffeine	0.14 [−0.06, 0.34]	0.174	4.6 [2–7]
Liquid form of caffeine	0.24 [−0.06, 0.54]	0.124	5.2 [3.7–6]
Males	0.16 [−0.02, 0,34]	0.081	5.3 [3–7]
Females	0.23 [−0.23, 0.69]	0.323	4.8 [2–6]
Athletes	0.23 [0.03, 0.42]	0.025	4.4 [2–6]
Non athletes	0.03 [−0.33, 0.40]	0.854	6.5 [6–7]
Countermovement jump	0.14 [−0.04, 0.32]	0.138	5.0 [3.7–7]
Sargent test	0.31 [−0.09, 0.70]	0.129	4.3 [2–6]

*SMD* standardized mean difference, *CI* confidence interval

**Fig. 5:**
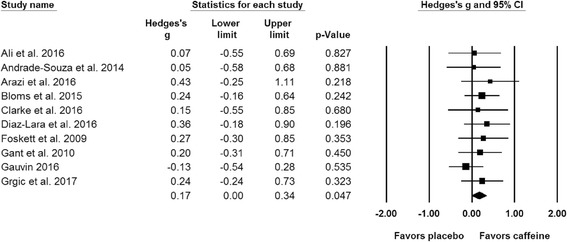
Forest plot showing differences between the effects of placebo and caffeine trials on measures of muscle power expressed as vertical jump height. The size of the plotted squares reflects the relative statistical weight of each study. The numbers on the *x*-axis denote the standardized mean differences expressed as Hedge’s g. The horizontal lines denote the respective 95% confidence intervals (CI)

The meta-analysis performed for muscle power indicated a significant difference (SMD = 0.17; 95% CI: 0.00, 0.34; *p* = 0.047) between the placebo and caffeine trials (Fig. 5). Results from all of the subgroup analysis can be found in Table 2.

The *I*^2^ statistic showed low heterogeneity for the studies assessing muscle strength and muscle power (*I*^2^ = 0.0; *p* = 0.981, and *I*^2^ = 0.0; *p* = 0.933, respectively). The analysis of funnel plots did not reveal substantial asymmetry for muscle strength or muscle power outcomes. The Trim-and-Fill method changed the pooled SMD for muscle power from 0.17 (95% CI: 0.00, 0.34) to 0.12 (95% CI: -0.01, 0.26). The Trim-and-Fill method did not have an impact on the pooled effect size for muscle strength outcomes.

The mean PEDro methodological quality score was 9.6, with the values for individual studies ranging from 8 to 10. Three studies [8, 14, 22] were categorized as being of “good methodological quality” (PEDro score = 8), while all other studies were classified as being of “excellent quality”.

## Discussion

The results of the meta-analysis show that caffeine may be an effective ergogenic aid for muscle strength and power. The pooled effects of caffeine on performance were small to medium. It is important to note that even small improvements in performance in some sports may translate to meaningful differences in competitive outcomes [27, 28]. A previous meta-analysis did not show a significant effect of caffeine supplementation on muscle strength [2], and the results of individual studies investigating caffeine’s effects on muscle power have not been previously pooled in a meta-analysis. Our novel results showing that caffeine may induce practically meaningful improvements in muscle strength and power can, therefore, be used to inform athletes, coaches, and sports nutritionists, as well as future research endeavors in this area, about the ergogenic potential of caffeine.

### Strength outcomes

#### Upper and lower body strength

The subgroup analysis indicated a significant increase in upper body, but not lower body strength, with caffeine ingestion. These results are somewhat unexpected, as Warren et al. [10] suggested that larger muscles, such as those of the lower body, have a greater motor unit recruitment capability with caffeine intake than smaller muscles, such as those of the arm. Motor unit recruitment, in addition to the reduced rate of perceived exertion and the central effects of adenosine on neurotransmission, arousal, and pain perception, are considered to be underlying mechanisms by which caffeine can enhance performance, although the exact mechanisms remain to be fully elucidated [29, 30]. Based on the current results, it may be surmised that caffeine is a useful ergogenic aid for achieving acute increases in maximal upper body strength. In the included studies, lower body maximal strength was evaluated using only leg press and squat (machine-based and free weight) tests. Two studies [4, 16] used a free weight exercise (barbell back squat), and both reported a significant increase in lower body strength. Warren et al. [10] concluded that caffeine ingestion might increase lower body isometric strength. Our findings do not indicate a strength increasing effect with caffeine ingestion for lower body dynamic strength. It is worth noting that in general, the included studies did not report on the reliability of their strength assessment, indicating potential reasons for the surprising findings for lower body strength. Further research is needed to examine the effects of caffeine on dynamic strength. Such studies may benefit from using a larger variety of dynamic lower body strength tests, as the current findings are mostly limited to a small selection of primarily machine-based tests.

#### Training status

The subgroup analysis for training status indicated no significant differences in maximal strength in trained (*p* = 0.076) and untrained individuals (*p* = 0.144). The meta-analysis of the three studies among untrained individuals was limited by small overall sample size (*n* = 32). It may be considered indicative that two of three individual studies reported significant differences in maximal strength with caffeine ingestion, but more individual studies on this topic are needed before drawing firm conclusions. Training status seems to play a significant role in response to caffeine intake in other forms of physical activity, such as swimming, with greater improvements observed in trained athletes [31]. However, it remains unclear whether the same applies to strength outcomes. More studies are needed before confidently drawing conclusions about the potential differences in effects of caffeine ingestion on muscle strength of trained and untrained individuals.

#### Sex

The subgroup analysis in males showed a significant improvement in strength with caffeine ingestion. The subgroup analysis for females was limited by small sample size, as only three studies [5, 12, 14] were found meeting the inclusion criteria. The landmark study by Goldstein et al. [5] reported a significant increase in the 1RM bench press in a cohort of resistance trained females. However, the effect size was very small (SMD = 0.07), thereby limiting the practical significance of the finding. Another study among female participants was performed by Sabblah et al. [14]. The researchers reported an SMD of 0.33 for increases in upper body strength with caffeine ingestion. However, the study employed a single-blind design and hence provided evidence of somewhat lower methodological quality compared to other studies. Additionally, the participants in the study from Sabblah et al. [14] exhibited lower levels of fitness than the participants in the study from Goldstein et al. [5], with marked disparities observed for 1RM strength (32 kg and 52 kg, respectively). None of the studies that included female participants controlled for the potential variability attributable to metabolic alterations across the menstrual cycle [32], which is a limitation of the current body of literature. Additional rigorously controlled studies are needed to provide clarity on the topic.

#### Caffeine form

The subgroup analysis indicated significant increases in strength after the ingestion of caffeine in the capsule form. The meta-analysis of the effects of the liquid form of caffeine included only three studies and did not report a significant effect. It is likely that the analysis was limited due to the small sample size (*n* = 50). Only one study [16] used caffeine in the form of a gel. Previous studies indicate that there are no practically meaningful pharmacokinetic differences between these routes of caffeine ingestion [33]; as such, it is unlikely that marked differences exist when comparing ergogenic effects of various forms of caffeine administration. Further investigations are needed for liquid forms of caffeine and others that have rarely or never been studied in this context, such as gum and gel.

#### Power outcomes

The meta-analysis supports caffeine as an effective ergogenic aid for achieving acute increases in muscle power expressed as vertical jump height. These results may have considerable applicability to many sports, including basketball and volleyball, in which muscle power and jumping ability are highly related to performance outcomes. The magnitude of acute improvement in vertical jump height found in the current analysis for a single caffeine ingestion is roughly equivalent to the effects of ~ 4 weeks of plyometric training [34]. The current analysis included only studies that used vertical jump as the power outcome; as such, it is possible that caffeine ingestion could produce somewhat different effects on other types of muscle power tests. However, a recent meta-analysis also showed a significant performance-enhancing effect of caffeine on the Wingate test, which is a common test of power [35]. Furthermore, most of the included studies used countermovement jump for assessing vertical jump; it remains to be explored whether the caffeine ingestion would produce different effects on other forms of vertical jumping. In addition, all of the included studies evaluated these effects in isolated conditions that may not accurately reflect in-game, sport-specific jumping tasks. More evidence may be needed to determine if the performance-enhancing effects of caffeine would transfer in the context of individual sports and/or team-sport matches [36].

While previous research [37] has shown an increase in countermovement jump height after ingestion of a caffeine-containing energy drink, it was unclear if the effect was attributable to the caffeine content or the presence of other substances, such as taurine. A recent meta-analysis on caffeinated energy drinks found a significant association between their taurine content and performance, but not between their caffeine content and performance [38]. As postulated by Bloms et al. [8], motor schema might play a role when assessing the association between caffeine and muscle power. Bloms et al. [8] tested the effect of caffeine on muscle power among a cohort of athletes and reported significant increases in jumping height. By contrast, Gauvin [26] reported no effects of caffeine ingestion on muscle power in a group of untrained men, with no previous experience in the exercise. The subgroup analysis for training status indicated a significant effect for athletes, but not for non-athletes. It may be suggested that future studies should control for this confounding factor by including only participants with or without previous experience in the task, or by performing initial familiarization sessions.

None of the remaining subgroup analysis showed a significant effect of caffeine. These results might be due to the small sample sizes in different subgroup analysis. More studies are needed before reaching conclusions about context-specific effects of caffeine. Furthermore, while the body of evidence evaluating effects of caffeine on muscle power is still limited; the current meta-analysis shows promising findings, but more studies are needed on this topic. Specifically, studies including different forms of vertical jumping and sport-specific jumping tasks, different population groups, larger sample sizes, and different doses and forms of caffeine are required.

#### Methodological quality

The PEDro scale showed good to excellent quality among the included studies, suggesting that the results of the current meta-analysis were not confounded by the inclusion of studies with poor research methodology. Only two studies [6, 25] reported receiving funding from parties that may have had commercial interest for conducting the research, so it is improbable that the overall results of the current study were significantly affected by financial bias. To further improve the quality of evidence, future studies should use a double-blind rather than a single-blind design and assess the effectiveness of the blinding. Only three studies [3, 22, 24] reported assessing the effectiveness of the blinding. This information is of importance as participants’ recognition of the caffeine trial may influence outcomes [39], because psychological effects of ‘expectancy’ and ‘belief’ might have an impact on performance [40]. In some studies, performance-enhancing responses were found with perceived ‘caffeine’ ingestion, when in fact, a placebo was consumed [41]. Future studies examining this topic should include a questionnaire of perception of the trials to prevent possible issues associated with such confounding.

While the inclusion of doctoral and master’s theses may be considered as a limitation of this review, their inclusion is supported by their high methodological quality scores. Therefore, the inclusion of such studies may be regarded as a strength rather than a limitation, as it would be inappropriate to omit high-quality contributions to the literature from a comprehensive systematic review. A limitation of the current review is the low number of studies included in the subgroup analysis. Secondly, a limitation is that no studies were found for age groups other than adolescents and young adults. The findings, therefore, pertain mainly to young individuals and cannot be generalized to other age groups. Furthermore, due to the high degree of inter-individual variability of effects [42], these results should be interpreted with caution when it comes to prescribing caffeine supplementation to individuals. Individuals should also assess their susceptibility to possible side effects as reported in the literature, such as tremor, insomnia, elevated heart rate, headache, abdominal/gut discomfort, muscle soreness, and inability to verbally communicate and stay focused. These side effects may be enhanced in naive caffeine users [3, 5], so extra precaution may be warranted in such individuals.

## Conclusion

Caffeine appears to provide significant ergogenic effects on muscle strength and power. The expression of strength in the form of 1RM is most specific to the sport of powerlifting but may translate to performance improvements in a variety of other strength-power sports. The effects of caffeine on muscle power may apply to athletes in a variety of sports in which jumping is a predominant activity that affects the sport-specific performance. Subgroup-analyses suggested that the effects of caffeine on strength may be more pronounced in upper body muscles, but further research on this topic is warranted. The results of the present meta-analysis are based on limited evidence, and thus need to be interpreted with caution. Future studies should explore the optimal dosage and form of caffeine for maximizing effects on strength and power. Finally, responses to caffeine ingestion have a high degree of inter-individual variability, and as such, the applicability of the current findings must be assessed on a case-by-case basis, based on the specific characteristics of the individual and the sports activity or other physical tasks.
